# Disseminated Infection Caused by *Eggerthella lenta* in a Previously Healthy Young Man: A Case Report

**DOI:** 10.1155/2012/517637

**Published:** 2012-12-17

**Authors:** Ahmad Salameh, Stephen A. Klotz, Tirdad T. Zangeneh

**Affiliations:** ^1^Department of Medicine, The University of Arizona Medical Center, University of Arizona, Tucson, AZ 85724, USA; ^2^Division of Infectious Diseases, Department of Medicine, The University of Arizona Medical Center, University of Arizona, Tucson, AZ 85724, USA

## Abstract

Anaerobic bacteria are the predominant normal flora of the mucous membranes which may cause life-threatening disseminated infections and are often difficult to culture from infected sites. *Eggerthella* (previously known as *Eubacteria* species) is an anaerobic, nonsporulating, nonmotile, Gram-positive rod that is found in the human colon and feces and has been isolated from various other clinical specimens. We report a case of complicated disseminated anaerobic bacterial infection with *Eggerthella lenta* in a healthy immunocompetent man causing multiple brain abscesses, liver abscesses, necrotizing pneumonia, and osteomyelitis of the left radial bone. He was successfully treated with empiric penicillin G and metronidazole.

## 1. Introduction

Anaerobic bacteria may cause life-threatening disseminated infections and are often difficult to culture from infected sites. We report a case of complicated disseminated anaerobic bacterial infection with *Eggerthella lenta*, previously known as *Eubacteria* species in a healthy immunocompetent man causing multiple brain abscesses, liver abscesses, necrotizing pneumonia, and osteomyelitis of the left radial bone. He was successfully treated with empiric penicillin G and metronidazole. 

## 2. Case Presentation

**Figure 1:**
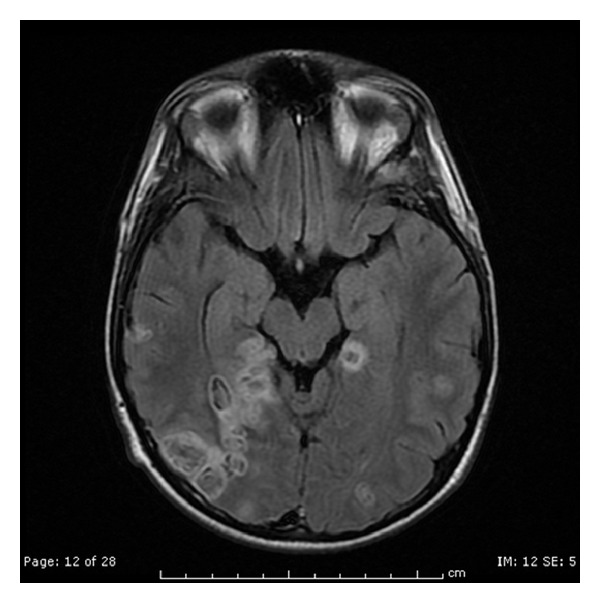
Multiple ring-enhancing and punctate areas of abnormal enhancement in the cerebral hemispheres bilaterally with some lesions demonstrating restricted diffusion.

**Figure 2:**
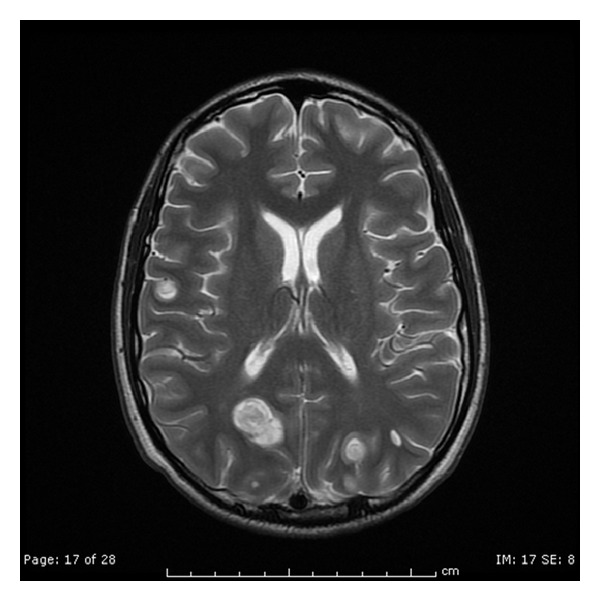
Multiple ring-enhancing and punctate areas of abnormal enhancement in the cerebral hemispheres bilaterally.

A 19-year-old male was transferred to our facility from a hospital in New York City where he had initially presented with fever, fatigue, weakness, loss of appetite, nonproductive cough, nausea, vomiting, and unintentional weight loss of about 20 pounds (9.07 Kilograms) over a two-week duration. His past medical history was significant for asthma and three episodes of pneumonia during childhood. His mother has common variable immunedeficiency (CVID) and antiphospholipid syndrome. The patient denied ingestion of raw food, exposure to animals, recent travel, or sick contacts and reported safe sex with one partner. He denied use of tobacco, alcohol, and injection drug use. On admission to the hospital in New York City he had a temperature of 40.0°C, heart rate of 126 beats per minute, respiratory rate of 20, blood pressure of 98/56, and oxygen saturation of 100% on room air. Physical examination was significant for crackles at the left base, hyperreflexia of the deep tendons of the upper and lower extremity with ankle clonus. Two sets of blood cultures were reported to be positive for *Eggerthella lenta. *Laboratory results were significant for a white-blood cell count of 15,700/mm^3^ with 78% neutrophils. His urinalysis and kidney function tests were within normal range, and liver function tests were mildly elevated. The chest X-ray was significant for a left lower lobe infiltrate. Computed tomography (CT) scan of the head did not reveal any significant findings. Magnetic resonance image (MRI) of brain showed multiple nodular densities in the white matter of the brain with edema. Enhancement of the leptomeninges was suggestive of meningitis (Figures 1 and 2). Computed tomography (CT) scan of the chest showed left a lower lobe consolidation with 1.0 cm cavitary lesion. A CT scan of the abdomen and pelvis showed multiple lesions in the right lobe of the liver and spleen likely representing abscesses, splenomegaly, small amount of ascites, and mild diffuse mesenteric edema. Ultrasound of the abdomen showed gallbladder sludge, small stones, and mild gallbladder wall thickening. Transesophageal echocardiogram showed normal left ventricular function and no evidence of endocarditis. Lumbar puncture was performed, and CSF analysis was normal. The patient was administered ceftriaxone and metronidazole.

The patient and family requested a transfer to our hospital due to the proximity to family and support care. He was then evaluated by the infectious disease services, and it recommended continuing on ceftriaxone and metronidazole. This was later switched to penicillin G 18,000,000 units daily and metronidazole 500 mg orally every 8 hours. Repeated testing for HIV, coccidioidomycosis, and syphilis was negative. Quantitative immunoglobulin titers were normal, and in light of his family history of CVID he underwent extensive testing for an immunodeficiency state by an immunologist and was found to have no underlying deficiencies. Two weeks later the patient was improving except for a complaint of left forearm pain and weakness. A left arm X-ray demonstrated osteomyelitis. Orthopedic surgery recommended continuing his antibiotics. The patient was continued on penicillin G and metronidazole for a total of 5 months. He has had follow-up MRI's of the brain at one month, three month, and six month which indicated marked reduction in the edema and abscesses involving the cerebral cortex, and the most recent study has shown marked improvement with only scarring. Repeat CT of the abdomen and pelvis was normal. Most recently he had laparoscopic cholecystectomy and found to have chronic cholecystitis, which may have been the source of his disseminated infection. During his last visit he reported returning to his normal state of health and an active healthy life.

## 3. Discussion

We report a case of complicated disseminated anaerobic bacterial infection caused by *Eggerthella lenta*, previously known as *Eubacterium *species. Members of the genus *Eggerthella* have been associated with disseminated infections as seen in this young man. Anaerobic bacteria are the predominant normal flora of the mucous membranes [1, 2]. *Eggerthella* is a bacterial genus of *Actinobacteria*, in the family *Coriobacteriaceae*. Members of this genus are anaerobic, nonsporulating, nonmotile, Gram-positive bacilli that grow singly, as pairs or in short chains. They are found in the human colon and feces and have been isolated from various clinical specimens including blood, abscesses, wounds, obstetric and genitourinary tract infections, and intra-abdominal infections [3, 4]. They have been associated with ulcerative colitis, Crohn's disease, Hepatobiliary diseases, abscesses, systemic bacteremia, malignancies, decubitus ulcers, diverticular abscess, appendicitis, pancreatic abscess, and pelvic inflammatory disease [5–8]. *Eggerthella lenta* is named for Arnold Eggerth, who first described the organism in 1935 [9, 10]. The genus *Eggerthella* was given to these bacteria in 1999 based on 16S rRNA sequence. Because of their fastidious nature, anaerobes are difficult to isolate and are often not recovered from infected sites. As PCR and sequencing techniques are becoming more readily available in clinical laboratories, 16S rRNA gene analysis will be a more reliable and practical approach to identify *Eggerthella* to the species level [6]. 

The mortality rate associated with *Eggerthella bacteremia* is about 20–40% in the previously reported cases, but larger prospective studies need to be done at multiple centers to determine the most effective treatment regimen. All *Eggerthella *species including *E. lenta, E. hongkongensis*, and *E. sinensis* have been associated with bacteremias of relatively high mortality [5, 6]. Absence of fever, hypothermia and stay in the ICU was associated with increased mortality per Venugopal et al. who reported 24 cases of *E. lenta* bacteremia [8]. 

*Eggerthella lenta* identification in blood culture should be considered serious and warrants evaluation of a source that can include skin and soft tissues, obstetric-genitourinary tract, and intra-abdominal infections. Our patient underwent laparoscopic cholecystectomy several months later after completion of antibiotic therapy due to the presence of gallbladder wall thickening and sludge on imaging and since abdominal imaging upon admission had features of cholecystitis. Neither 16S rRNA sequencing nor antibiotic susceptibilities were performed on the cultures initially done in New York, and the liver abscess aspirate was negative for microorganisms on stain and culture. In a recent study by Lee et al. antimicrobial susceptibilities of 17 blood isolates for *Eggerthella, Paraeggerthella, *and* Eubacterium* species at a University Hospital in Taiwan, from 2001 to 2010 were reported [11]. In this study all isolates were susceptible to ampicillin-sulbactam, metronidazole, imipenem, and meropenem.Many hospitals do not perform antimicrobial susceptibilities on anaerobic organisms on a regular basis; however, for successful treatment of serious life threatening infections clinical specimens may be sent to other institutions capable of performing such task.
